# Expression of Indoleamine 2,3-Dioxygenase Induced by IFN-γ and TNF-α as Potential Biomarker of Prostate Cancer Progression

**DOI:** 10.3389/fimmu.2018.01051

**Published:** 2018-05-29

**Authors:** Irina Banzola, Chantal Mengus, Stephen Wyler, Tvrko Hudolin, Gabriele Manzella, Alberto Chiarugi, Renzo Boldorini, Giovanni Sais, Tobias S. Schmidli, Gabriele Chiffi, Alexander Bachmann, Tullio Sulser, Giulio C. Spagnoli, Maurizio Provenzano

**Affiliations:** ^1^Oncology Research Unit, Department of Urology, University Hospital of Zurich, Zurich, Switzerland; ^2^Department of Oncology and Children’s Research Center (CRC), University Children’s Hospital, Zurich, Switzerland; ^3^Department of Biomedicine, University Hospital of Basel, Basel, Switzerland; ^4^Department of Urology, University Hospital of Basel, Basel, Switzerland; ^5^Department of Preclinical and Clinical Pharmacology, University of Florence, Florence, Italy; ^6^Department of Health Science, School of Medicine, Università degli Studi del Piemonte Orientale, Novara, Italy; ^7^CNR Institute of Translational Pharmacology, Rome, Italy

**Keywords:** indoleamine 2,3-dioxygenase, IDO, prostate cancer, inflammation, prostate cancer prognosis

## Abstract

Inflammation has been suggested to play an important role in onset and progression of prostate cancer (PCa). Histological analysis of prostatectomy specimens has revealed focal inflammation in early stage lesions of this malignancy. We addressed the role of inflammatory stimuli in the release of PCa-specific, tumor-derived soluble factors (PCa-TDSFs) already reported to be mediators of PCa morbidity, such as indoleamine 2,3-dioxygenase (IDO) and interleukin (IL)-6. Inflammation-driven production and functions of PCa-TDFSs were tested “*in vitro*” by stimulating established cell lines (CA-HPV-10 and PC3) with IFN-γ or TNF-α. Expression of genes encoding IDO, IL-6, IFN-γ, TNF-α, and their receptors was investigated in tumor tissues of PCa patients undergoing radical prostatectomy, in comparison with benign prostatic hyperplasia (BPH) specimens. IFN-γ and TNF-α-treatment resulted in the induction of IDO and IL-6 gene expression and release in established cell lines, suggesting that the elicitation of PCa-TDSFs by these cytokines might contribute to progression of cancer into an untreatable phenotype. An analysis based on timing of biochemical recurrence revealed the prognostic value of IDO but not IL-6 gene expression in predicting recurrence-free survival in patients (RFS) with PCa. In addition, a urine-based mRNA biomarker study revealed the diagnostic potential of IDO gene expression in urines of men at risk of PCa development.

## Introduction

Chronic inflammation is considered an “enabling” characteristic of human cancers (1), although its prognostic significance in malignancies of different histological origin is debated (2). Histological analysis of prostate cancer (PCa) samples consistently indicates that inflammation might play a key role in the progression of this tumor (3). PCa is more frequent in demographic groups with high prevalence of prostatic inflammation (4), with a higher risk for obese men (5, 6). In addition, prostate adenocarcinoma outgrowth is frequently detected in tissue areas adjacent to chronic inflammation (3, 4). However, other studies suggest that histologically detectable prostate inflammation associates with decreased PCa risk (7, 8).

Previous studies by us and others indicate that genes encoding pro-inflammatory cytokines are expressed to significantly higher extents in PCa than in benign prostatic hyperplasia (BPH) tissue specimens (9–11).

Chronic inflammation frequently results in the establishment of an immunosuppressive microenvironment, particularly in cancer tissues (12, 13). In this context, pro-inflammatory cytokines might promote survival and proliferation of tumor cells and, paradoxically, subvert adaptive immune responses (13). Numerous studies indicate that chronic immune-stimulation may result in immune tolerance. Overstimulated T cells do express “immunological checkpoint” receptors whose triggering by specific ligands results in their anergy. Notably, the expression of some ligands of these receptors is typically induced by IFN-γ. These concepts provide the basis for the use of specific biologicals in cancer treatment and represent an important link between the potential immunogenicity of tumor microenvironment and the neutralization of immune responses (14).

IFN-γ and TNF-α are typically produced in the context of ongoing immune responses against infectious and tumor challenges. However, TNF-α has also been proposed as PCa marker (15). TNF-α might exert pro- or antiapoptotic effects, depending on cell or tissue types and experimental conditions (16, 17). High levels of TNF-α lead to tumor cell necrosis and apoptosis (18), and mediate immune cytotoxicity and production of inflammatory cytokines (19). In contrast, low-dose paracrine TNF-α production in tumor areas may support chronic inflammation and cancer progression (20).

Regarding IFN-γ, PCa cells have been shown to be poorly sensitive to its cytotoxic effects (21). Furthermore, it has been suggested that IFN-γ might induce immune-suppressive effects in PCa (22, 23).

In this study, we have comparatively analyzed the expression of genes encoding IFN-γ, TNF-α, and their receptors in PCa and in BPH tissues. Moreover, we have investigated the ability of these cytokines to induce in PCa cells the production of factors promoting tumor progression through the inhibition of adaptive immune responses (24) and/or the activation of alternative pathways for the androgen receptor (25). Finally, we have addressed the prognostic significance of factors induced by IFN-γ and TNF-α in PCa cells and their potential predictive power when detected in urine of individuals at risk of developing this malignancy.

## Materials and Methods

### Patients

We studied two case series of 97 consecutive PCa patients undergoing radical prostatectomy and 35 consecutive BPH patients undergoing conventional transurethral resection of the prostate (TUR-P) enrolled in the Department of Urology of the University Hospital of Basel, Switzerland, from 2007 to 2011. Clinicopathological parameters [prostate-specific antigen (PSA) levels, tumor stage, and Gleason score] were assigned according to EAU guidelines for PCa (uroweb.org/guideline/prostate-cancer/). If a PCa patient had a tumor whose stage at diagnosis could not be assessed, the Gleason score at biopsy was reported. BPH urinary obstructive symptoms and acute urinary retention were evaluated according to the international prostate symptom score. Biochemical recurrence (BR) was documented in 21 of 97 patients (21.6%) over a median follow-up of 23 months (range, 3–63 months). Local ethics committee approval and written informed consent from patients were obtained in accordance with the requirements of the Ethical Committee of Basel-Stadt and Basel-Land (EKBB, Ref. Nr. EK: 176/07). For the detection of indoleamine 2,3-dioxygenase (IDO) and interleukin (IL)-6 gene expression in urines of individuals at risk of developing PCa, we collected urines of 27 consecutive patients before they underwent fine needle biopsy for diagnostic/prognostic purposes (KEK-ZH-NR: 2010-0081/0).

### Cell Culture

Established human PCa cell lines CA-HPV-10 [immortalized cell line from high-grade adenocarcinoma; American Type Culture Collection (ATCC)^®^CRL-2220] and PC3 (lumbar metastasis; ATCC^®^CRL-1435) were purchased from the ATCC (LGS Standards). Cells were cultured according to Russel and Kingsley guidelines (26). In particular, keratinocyte serum-free medium, supplemented with 50 µg/ml bovine pituitary extract, and Roswell Park Memorial Institute 1640 medium, supplemented with 10% fetal bovine serum, were used. All experiments were performed with cells at passage 9 or below. Cells were regularly tested for mycoplasma contamination by PCR (LookOut^®^ mycoplasma qPCR detection kit, Sigma-Aldrich, Switzerland), prior to experimental procedures. Cells were cultured in T75 flasks and, when reaching 80% confluence, were stimulated with either IFN-γ or TNF-α, at a concentration of 300 U/ml (eBioscience, San Diego, CA, USA) or left unstimulated. Cells were harvested at different time points (6, 12, and 24 h for PC3 and 6, 12, 24, 48, and 72 h for CA-HPV-10). For inhibition tests, cells stimulated with IFN-γ or TNF-α (300 U/ml) or left unstimulated were cultured for 24 h in the presence of ruxolitinib (INCB018424; 1 µM) (27), SB202190 (20 µM) (28), LY294002 (50 µM) (29) (Selleckchem.com, Houston, TX, USA), or PDTC (10 µM; Sigma-Aldrich) (30) or without inhibitors. Cells were then collected by trypsinization (trypsin-EDTA, Invitrogen, Carlsbad, CA, USA) and used for gene expression analysis.

### Gene Expression Analysis

Whole-mount prostatectomy sections of patients’ tissue specimens were analyzed for the presence of tumors by experienced pathologists. Total RNA was extracted from tumor tissues by the RNeasy^®^ MiniKit (Qiagen, Basel, Switzerland), treated with deoxyribonuclease I (Invitrogen), and reverse transcribed by using M-MLV Reverse Transcriptase (Invitrogen). Quantitative gene amplification (qRT-PCR) was performed by using an ABI Prism 7500 FAST sequence detection system with the TaqMan^®^ Universal PCR Master Mix and primers and probes provided by Assays-on-Demand, Gene Expression Products (Life Technologies, Forster City, CA, USA). Normalization of gene expression was performed by using GAPDH gene as reference. The 2^−ΔΔ^*^Ct^* method was used to compute the fold change in the expression of genes of interest when compared with GAPDH gene expression as baseline. Values ≤10^−7^ were considered undetectable. For cell line analysis, cells were cultured, stimulated, and harvested as described above, and gene expression was similarly evaluated.

### Measurement of Tryptophan and Kynurenine Concentrations

Measurement of tryptophan and kynurenine concentrations in supernatants of cytokine-treated and -untreated cell lines, collected at defined time points was performed by high-pressure liquid chromatography (HPLC) and fluorimetric detection, as previously described (31). Supernatants were deproteinized by mixing with an equal volume of 10% (w/v) trichloroacetic acid. Measurement of tryptophan by HPLC separation was performed with a reverse-phase column (Spherisorb S5 ODS2, 25 cm) and a mobile phase (1 ml/min flow rate) composed of 5% acetonitrile, 100 mM phosphate buffer, pH 3.6, and 1 mM EDTA. Detection was performed with a Perkin-Elmer (Foster City, CA, USA) model LC 240 fluorimeter. The excitation and emission wavelengths were 313 and 420 nm, respectively. Kynurenine was measured by HPLC and UV detection. Briefly, HPLC separation was obtained with a reverse-phase column (Spherisorb S5 ODS2, 10 cm) and a mobile phase (1 ml/min flow rate) composed of 2% acetonitrile, 0.1 mM ammonium acetate, and 100 mM acetic acid. Kynurenine was detected at 365 nm with a UV detector (Perkin-Elmer model LC 90).

### ELISA Assays

Supernatants of cytokine-treated and -untreated cell lines were collected at defined time points, centrifuged for 10 min at 1,280 rpm to discard the cell debris, and subsequently stored at −80°C. IL-6 release was quantified by using a commercially available ELISA kit (eBioscience) according to the manufacturer’s instructions.

### Flow Cytometry Analysis

To evaluate expression of IFN-γ and TNF-α receptors, cells were stained with fluorescein isothiocyanate (FITC)-conjugated anti-interferon gamma receptor 1 (IFNGR1) mAb, allophycocyanin (APC)-conjugated anti-IFNGR2 mAb (R&D, Minneapolis, MN, USA), FITC-conjugated anti-tumor necrosis factor receptor 1 (TNFR1) mAb (Abcam), and phycoerythrin (PE)-conjugated anti-TNFR2 mAb (BD Pharmingen, San Diego, CA, USA). Data were acquired on a FACSCalibur^®^ cytometer (Becton Dickinson, Franklin Lakes, NJ, USA) and analyzed by FlowJo software.

### Immunohistochemistry

Representative 5-μm-thick sections of prostatic adenocarcinoma were cut from formalin-fixed and paraffin-embedded specimens and evaluated by immunohistochemistry with primary monoclonal antibodies anti-TNF-α (clone 52B83) and anti-IFN-γ (clone LLOSZ), using a Ventana Benchmark instrument (Ventana, Vreden, Germany). As positive controls, sections from reactive lymph nodes were used.

### Scratch Wound Healing Assay

Cytokine-treated and -untreated cell lines were seeded in triplicates in six-well plates and grown until confluent. Identical scratches were generated both vertically and horizontally with a 200-µl sterile pipette tip, creating a cross-shaped empty area. Cellular debris were removed by washing with PBS. Cells were center-imaged at 5× magnification, using a Zeiss Axiovert 200 M microscope equipped with a Zeiss AxioCam MRm camera with maximum contrast (Carl Zeiss AG, Feldbach, Switzerland; software: Axio Vision Rel. 4.7). For cell stimulation, either IFN-γ or TNF-α were added to the wells at a final concentration of 300 U/ml. The closure rate of the empty area was analyzed after 12 and 24 h. Data were evaluated with T-scratch software with default parameter setting.

### Boyden Chamber Invasion Assay

Cell invasion assays were performed in a Boyden chamber system using the Chemicon Cell Invasion Assay Kit (Millipore, Billerica, MA, USA), according to the manufacturer’s protocol. Cells were seeded in a 24-well plates (200,000 cells/well) in serum-free conditions in an ECMatrix-coated insert and stimulated with either cytokine afterward. Complete medium (10% FCS) was used as a chemoattractant and was placed in the bottom chamber of the plate. Cells were allowed to migrate for 48 h. Invading cells were then collected and quantified using the CyQuant GR dye (Millipore). All experiments were conducted in duplicate and repeated three times. Results are expressed as percentages of invading cells compared with unstimulated controls.

### Urine Processing

For urine tests, 20 ml of urine collected before biopsy were centrifuged at 2,000 rpm for 10 min. Total RNA was extracted from cell pellet by adding 700 µl of lysis solution to the pellet (RNA Aqueous isolation kit, Ambion). The elution step was performed by adding twice 50 µl of DNAse/RNAse free water (final volume 100 µl). For reverse transcription, 30 µl of RNA were added to 30 µl of a mix prepared with the High Capacity cDNA Reverse Transcription Kits (Applied Biosystems). The IDO assay (Custom TaqMan primers and probe design; Applied Biosystems) was designed to cover the exon–exon junction between exon 9 and 10. Primers were designed to allow a melting temperature between 58 and 61°C, with an optimal length of 20 bp and CG content between 30 and 80%. The probe was designed not to start with G and in order to have a melting temperature 10°C higher than the one of the primers. A 3′ minor grove binder-probe [non-fluorescent quencher fitting the 5′ 6-carboxy fluorescein (FAM) spectral qualities] was used. Primers and probe were used at a final concentration of 400 and 200 nM, respectively. TaqMan assay sequences are described in patent PCT/EP2017/076169. Gene quantification was performed by qRT-PCR as described above.

### Statistical Analysis

Statistical analyses were performed with GraphPad Prism (v5.1) and SPSS (v23). For the analysis of cell line data, the Mann–Whitney *U* test was used to compare the mean expression of specific genes or protein release for independent samples. For the analysis of patient data, non-parametric tests for gene expression levels (Mann–Whitney *U* test), correlation of gene expression between two factors (Spearman’s ρ), and frequency of expression between two groups of patients (Pearson χ^2^) were evaluated. For contingency table analysis, Fisher’s exact test was performed. For survival analysis, estimates of recurrence-free survival (RFS) were calculated by the Kaplan–Meier method and compared by the log rank test. Endpoint was time from surgery to BR, with censoring of data from patients who were BR negative at the time of their last follow-up visit. A univariate Cox regression analysis was performed to evaluate the hazard ratio (HR) for recurrence in IDO- and IL-6-positive patients. A multivariate Cox regression analysis was used to determine the independent prognostic relevance of both factors for the analyzable patients. For all tests, differences with *P* ≤ 0.05 were considered significant.

## Results

### IFN-γ and TNF-α Gene Expression in PCa Tissues

IFN-γ and TNF-α play key roles in acute and chronic inflammation. To obtain an insight into their potential relevance in PCa, we analyzed the expression of genes encoding these cytokines in tissue specimens from 64 patients collected after radical prostatectomy (RP) for primary PCa. Tissue specimens from patients treated for BPH (*n* = 35) were tested as controls.

**Figure 1:**
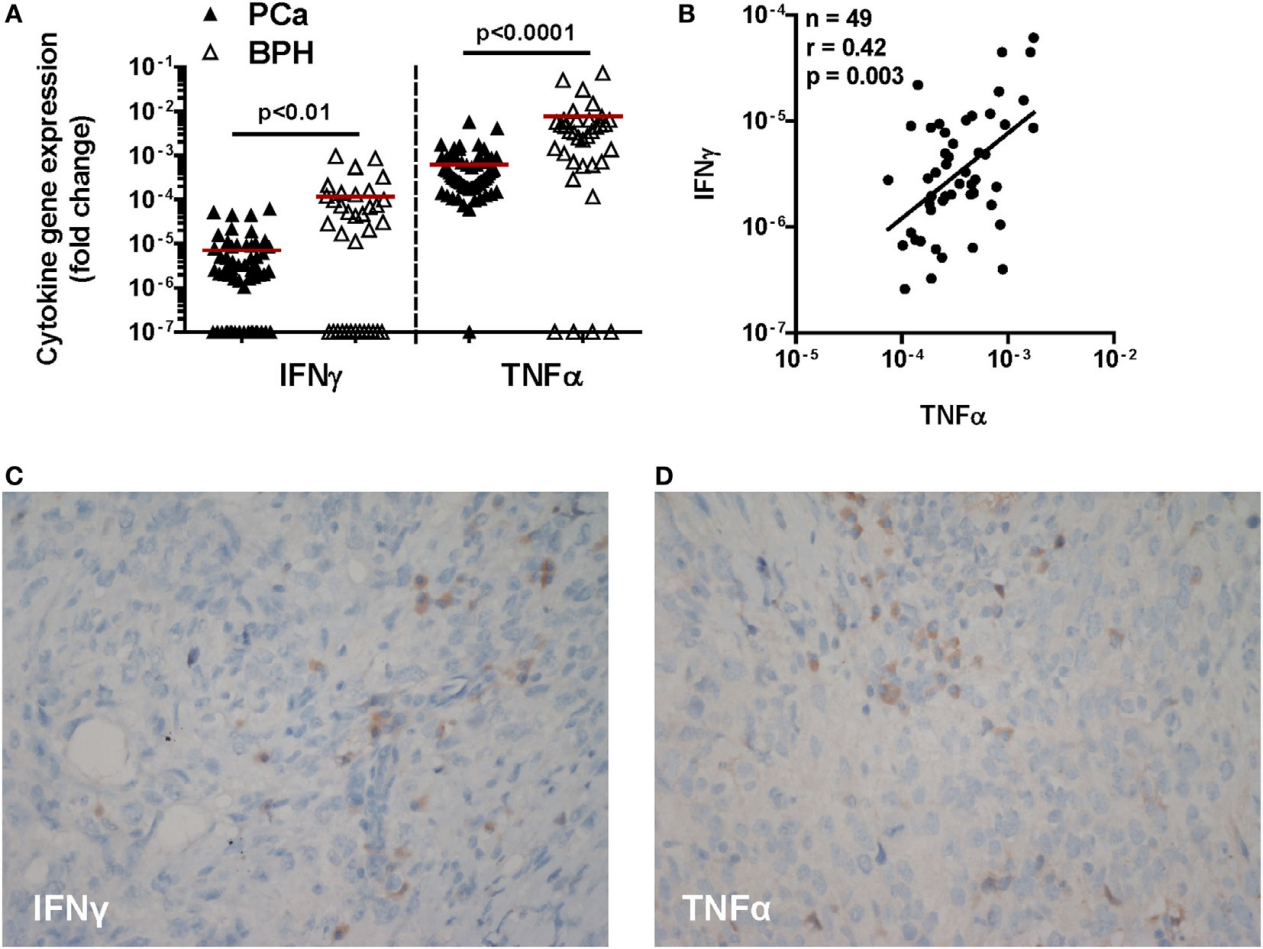
Expression of IFN-γ and TNF-α genes and immunohistochemical cytokine detection in prostate cancer (PCa) tissues. **(A)** Expression of IFN-γ and TNF-α genes in PCa (black triangles) and benign prostatic hyperplasia (BPH) (open triangles). A higher expression of both cytokine genes in BPH than in PCa tissue specimens was observed (Mann–Whitney *U* test, IFN-γ *p* < 0.01 and TNF-α *p* < 0.0001). The 2^−ΔΔ^*^Ct^* method was used to compute fold changes in gene expression for both cytokines, setting GAPDH gene expression equal to 1. Values ≤10^7^ were considered undetectable. **(B)** Fold changes in IFN-γ gene expression in either BPH or PCa tissue specimens were plotted against fold changes in TNF-α gene expression in same specimens. A significant correlation was only observed in PCa (Spearman rank correlation coefficient, *r* = 0.42; *p* < 0.01). **(C,D)** A representative image by immunohistochemistry (original magnification 200×; slight hematoxylin conterstaining) of a poorly differentiated prostatic adenocarcinoma (Gleason score 9) tissue specimen stained with **(C)** IFN-γ or **(D)** TNF-α.

*Ex vivo* analysis showed that IFN-γ and TNF-α genes are expressed with similar frequency in PCa and BPH. In particular, IFN-γ gene was expressed in 78 and 63% of cases, respectively (*p* = 0.1), whereas TNF-α gene expression was detected in 97% of PCa tissues and 90% of BPH tissues (*p* = 0.1) (Table 1). However, expression levels of both genes were significantly higher in BPH than in PCa specimens (IFN-γ, *p* < 0.01; TNF-α, *p* < 0.0001) (Figure 1A).

**Table 1:** Distribution of tumor-derived soluble factors (TDSFs) gene expression in prostate cancer (PCa) and benign prostatic hyperplasia (BPH) tissues.

TDSFs	Pca; *n* = 64	BPH; *n* = 35	*p*-Value^a^
IFNγ	50 (78%)	22 (63%)	0.1
TNFα	62 (97%)	31 (90%)	0.1
IDO	58 (91%)	18 (51%)	<0.0001
IL-6	47 (73%)	18 (51%)	0.03

*^a^Two-sided Pearson χ^2^ test*.

Remarkably, gene expression levels of these two pro-inflammatory cytokines were significantly correlated with each other in PCa (*n* = 49; *r* = 0.42; *p* < 0.01) (Figure 1B), but not in BPH tissues (*n* = 35, *r* = 0.2; *p* = 0.2, data not shown).

At the protein level, cancer cells showed only slight and focal cytoplasmic IFN-γ specific immunostaining and slight diffuse positivity along cell membranes to anti-TNF-α antibody. In contrast, interstitial inflammatory cells were intensely positive for both antibodies (Figures 1C,D).

Thus, consistent with putatively ongoing inflammation states, expression of both IFN-γ and TNF-α is detectable at the gene and protein level in both PCa and BPH tissues.

### PCa Cells Express IFN-γ and TNF-α Receptors

**Figure 2:**
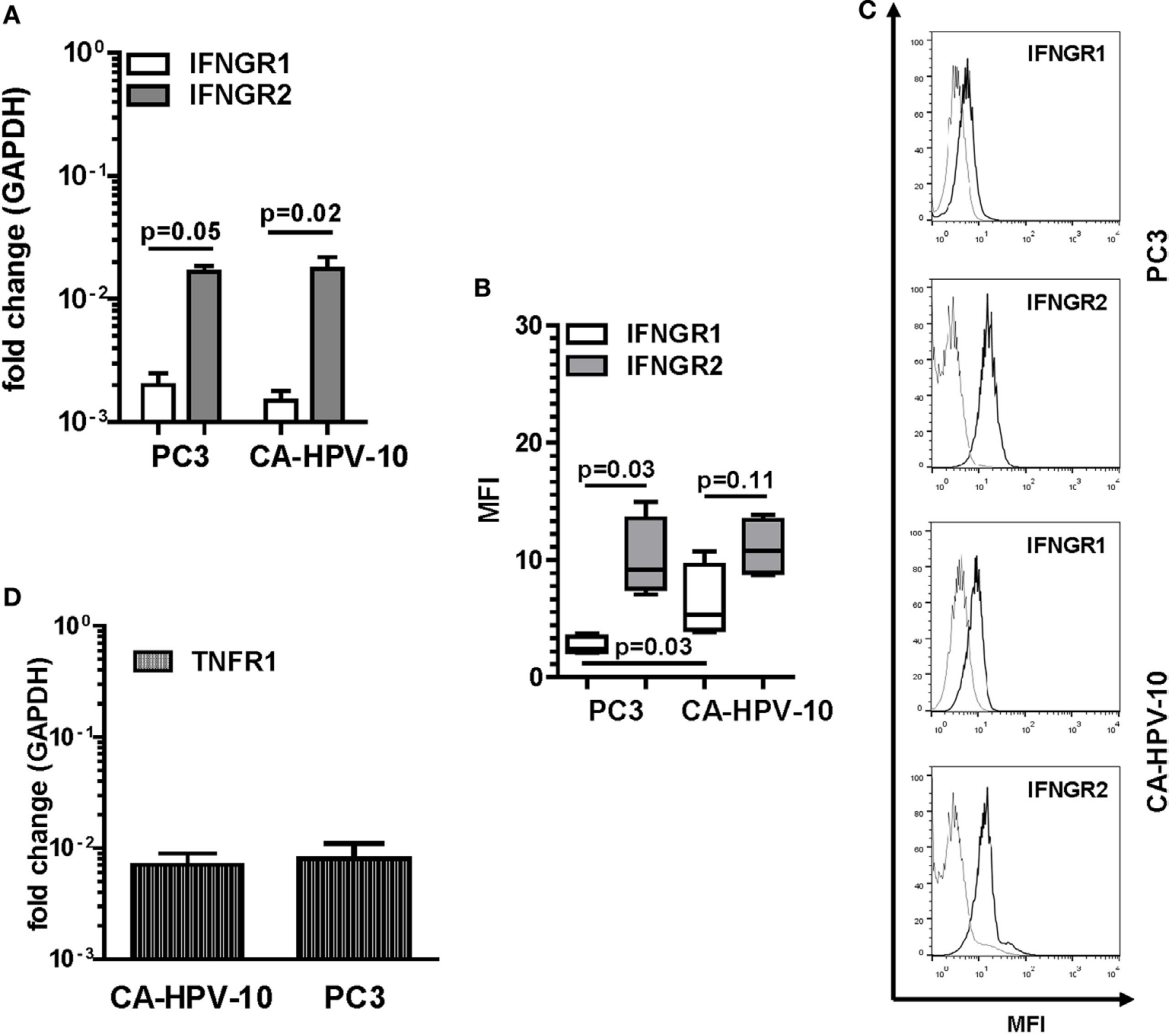
Interferon gamma receptors (IFNGRs) and tumor necrosis factor receptor 1 (TNFR1) gene and protein expression in prostate cancer (PCa) cell lines. **(A)** Gene expression of IFNGR1 (white bars) and IFNGR2 (gray bars) in PC3 and CA-HPV-10 cell lines. A higher expression of IFNGR2 than of IFNGR1 was observed in both cell lines (Mann–Whitney *U* test, PC3 *p* = 0.05 and CA-HPV-10 *p* = 0.02). **(B)** Mean fluorescence intensity (MFI) by fluorescent-activated cell sorting of IFNGR1 (white boxes) and IFNGR2 (gray boxes) specific staining in PC3 and CA-HPV-10 cell lines. **(C)** Representative overlaid histograms refer to MFI of IFNGR1 and IFNGR2, when compared with isotype staining. IFNGR2 was expressed to higher extents than IFNGR1 only in PC3 (Mann–Whitney *U* test, *p* = 0.03). IFNGR1 was expressed to a higher level in CA-HPV-10 than in PC3 cells (Mann–Whitney *U* test, *p* = 0.03). **(D)** TNFR1 gene expression in PC3 and CA-HPV-10 cell lines. No significant differences were observed between cell lines (Mann–Whitney *U* test).

IFN-γ and TNF-α expression in tissues urged us to explore the direct effects of these cytokines on PCa cells. As “*in vitro*” model, we chose two established cell lines originating from localized, CA-HPV-10, and metastatic, PC3, cancers. In preliminary experiments, we evaluated the expression of IFN-γ and TNF-α receptors in these cells. CA-HPV-10 and PC3 cells expressed IFNGR1 cytokine binding chain and, to higher extents, IFNGR2 accessory chain, at both gene (Figure 2A) and protein levels (Figures 2B,C). Furthermore, expression of the gene encoding TNFR1, the main receptor for soluble TNF-α (32), was detectable in both PCa cell lines (Figure 2D). However, TNFR1 protein expression was modest, and almost negligible in both cell lines, as assessed by Western blot and flow cytometry tests (data not shown).

### Pro-Inflammatory Stimuli Enhance the Expression of Tumor-Derived Soluble Factors Involved in PCa Progression

IFN-γ and TNF-α receptor expression by PCa cells suggested that these cytokines could directly stimulate tumor cells. To address this issue, we analyzed the ability of IFN-γ and TNF-α to modulate the expression of a panel of genes encoding a variety of factors known to be produced by tumor cells and of potential relevance in tumor progression and inhibition of immune responses (33). These genes were expressed to different extents in the PCa cells under investigation. In particular, *IDO, IL10*, and *HGF* genes were constitutively expressed in PC3, but not in CA-HPV-10 cells whereas expression levels of *IL6, ANG1, eNOS*, and *PDGF-BB* genes were 2.5-fold higher in PC3 than in CA-HPV-10 cells. On the other hand, *IL1*β and *MMP-9* were detected at higher levels in CA-HPV-10 than in PC3 (>2.5-fold). Additional genes, including *TGF*β*1, VEGF-A, -C*, and *-D, CCR7, MMP-2, ARG II, i-NOS, ANG2*, and *FRAG1*, were similarly expressed in both cell lines. It is noteworthy that *TNF-*α gene was constitutively expressed in both cell lines, whereas *IFN-*γ was undetectable (Table 2).

**Table 2:** Constitutive and cytokine-stimulated expression of genes encoding tumor-derived soluble factors involved in prostate cancer (PCa) progression (33).

	Constitutive expression over 24 h			Enhanced expression upon			
				IFNγ		TNFα	
	CA-HPV-10	PC3		CA-HPV-10	PC3	CA-HPV-10	PC3
TGFβ1			TGFβ1				
IL1β			IL1β				
IL-6			IL-6A				
IL10			IL10				
VEGF-A			VEGF-A				
VEGF-C			VEGF-C				
VEGF-D			VEGF-D				
CCR7			CCR7				
MMP2			MMP2				
MMP9			MMP9				
IDO			IDO				
ARG II			ARG II				
eNOS			eNOS				
iNOS			iNOS				
ANG 1			ANG1				
ANG 2			ANG2				
FRAG1			FRAG1				
HGF			HGF				
PDGF-BB			PDGF-BB				
TNFα			TNFα				
IFNγ			IFNγ				

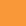
 *Constitutive gene expression, as compared with β-actin gene expression as 1,range 1.5- to 2.5-fold. Expression <1.5-fold no-reproducible gene*.

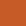
 *Significant relative gene expression (ΔΔ*Ct* ≥ 2.5-fold)*.

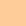
 *Negligible expression (≤10^−7^)*.

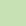
 *No relevant fold increase upon TNF-α or IFN-γ stimulation*.

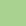
 *Gene expression at 6 hours over the constitutive expression (ΔΔ*Ct* ≥ 2.5-fold)*.

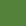
 *Gene expression at 12 hours over the constitutive expression (ΔΔ*Ct* ≥ 2.5-fold)*.

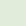
 *Negligible expression (≤10^−7^)*.

### Differential Effects of IFN-γ and TNF-α Stimulation of Established PCa Cell Lines

**Figure 3:**
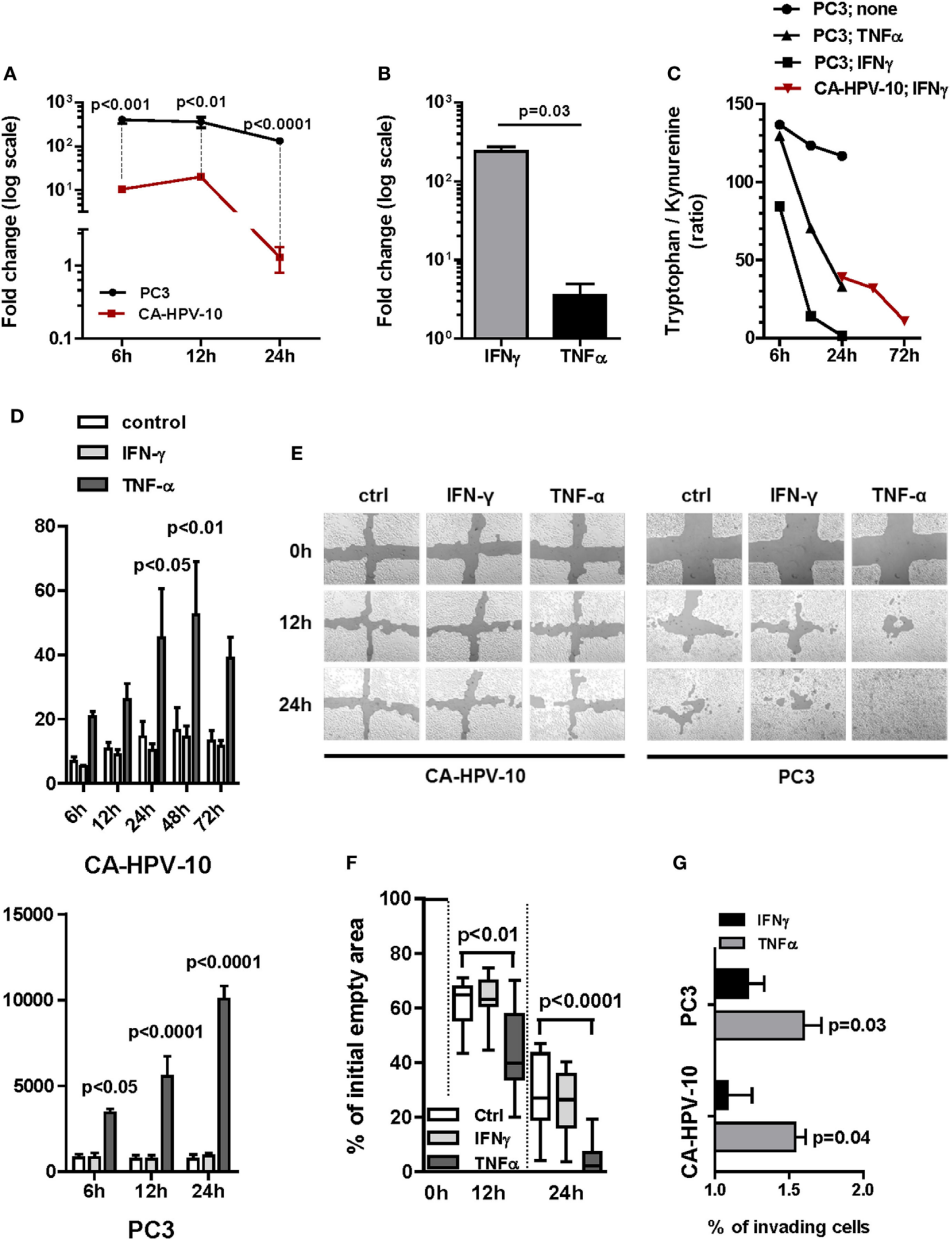
Gene expression of indoleamine 2,3-dioxygenase (IDO) and interleukin (IL)-6 and their functional activity in cell lines. **(A)** IDO gene expression upon IFN-γ induction of either PC3 (black lines and circles) or CA-HPV-10 (red lines and squares) cell lines was calculated as twofold higher than its constitutive expression in PC3. The cytokine induced a significantly higher IDO fold increase in PC3 than in CA-HPV-10 cell lines at 6, 12, and 24 h (Mann–Whitney *U* test). **(B)** IDO gene expression upon TNF-α stimulation was 2-fold less than upon IFN-γ. **(C)** IDO activity was measured as the tryptophan/kynurenine ratio detected in the cell supernatants. IFN-γ (black squares) induced a stronger enzymatic activity of IDO than did TNF-α (black triangles) in PC3, while to a lesser extent in CA-HPV-10 (red triangles). The test was carried out with pooled supernatants from three different experiments for each cell line under investigation cultured in the indicated conditions. Due to the negligible expression of IDO at constitutive level in CA-HPV-10 (Table 2 for gene expression at constitutive level), we did not measure tryptophan/kynurenine ratio in the supernatant of unstimulated CA-HPV-10 (control). **(D)** Protein release (pg/ml) of IL-6 in supernatant of CA-HPV-10 (above) or PC3 (below) cell lines treated with IFN-γ (light gray bars) or TNF-α (dark gray bars), when compared with untreated cell lines (white bars). Overall, TNF-α induced the highest release of the cytokine, compared with IFN-γ induction, in both cell lines at all-time points tested (Mann–Whitney *U* test). **(E)** Cell migration was assessed by a scratch wound healing assay, as shown in one representative experiment out of three by confocal microscopy. In our experimental conditions, CA-HPV-10 showed very limited migratory capability, which was unaffected by either cytokine, while PC3 showed a constitutively high motility, which was significantly enhanced by TNF-α (12 h, *p* < 0.01; 24 h, *p* < 0.001). **(F)** Boxes and whiskers representing migration potential of PC3 cells based on the quantification in percentage of empty area when compared with time 0 (100% empty area) upon treatment with IFN-γ (light gray boxes) or TNF-α (dark gray boxes) at time points of 12 h (*p* < 0.01) and 24 h (*p* < 0.0001; two-way ANOVA test) when compared with control. Data are expressed as averages of triplicates for each condition. **(G)** For the Boyden chamber cell invasion analysis, cells were allowed to invade the membrane matrix for 48 h. The invading cells were then collected and quantified using the CyQuant GR dye to measure the invasiveness of both CA-HPV-10 and PC3 after treatment with IFN-γ (black bars) or TNF-α (gray bars). We observed that TNF-α strongly increased the invasiveness of both CA-HPV-10 (54.08 ± 0.12%, *p* = 0.04) and PC3 (59.67 ± 0.21%, *p* = 0.03), when compared with IFN-γ-treated cells (CA-HPV-10, 8.08 ± 0.29%; PC3, 21.96 ± 0.19%). The results were calculated as percentages of invading cells compared with unstimulated controls. All experiments were conducted in duplicate and repeated three times.

IFN-γ and TNF-α failed to modulate the expression of the large majority of the genes under investigation (Table 2). However, IFN-γ stimulation significantly enhanced IDO gene expression (>400-fold after 6 h) in PC3 cells (Figure 3A, black lines). In addition, IFN-γ induced upon 6 h stimulation the *de novo* expression of IDO gene in CA-HPV-10 cells at levels 10-fold higher than those constitutively observed in PC3 cells (Figure 3A, red lines). TNF-α stimulation also significantly enhanced IDO expression in PC3 cells 3.6-fold above constitutive levels, although to a lesser extent, when compared with IFN-γ induction (*p* = 0.03; Figure 3B). However, TNF-α failed to induce *de novo* IDO gene expression in CA-HPV-10 cells.

To validate these findings, we evaluated IDO functional activity by testing tryptophan/kynurenine ratios in culture supernatants. In agreement with gene expression results, consistent with increased IDO functional activity, a marked decrease in tryptophan/kynurenine ratio was observed in supernatants from IFN-γ and TNF-α treated PC3 cells and from IFN-γ stimulated CA-HPV-10 cells. Notably, in PC3 cells, IDO activity was increased upon 6 h IFN-γ induction whereas upon TNF-α stimulation, it was significantly detectable only after 12 h. In keeping with qRT-PCR data indicating *de novo* IDO gene expression upon IFN-γ stimulation, increased IDO-related enzymatic activity in CA-HPV-10 cells was detectable with delayed kinetics after 24–72 h (Figure 3C). As expected, TNF-α treatment of CA-HPV-10 cells was ineffective (negligible expression in Table 2).

On the other hand, TNF-α, but not by IFN-γ, induced overexpression of IL-6 gene in both PC3 and CA-HPV-10 cells (Table 2) and, in accord with gene expression data, IL-6 release in supernatants of IFN-γ and TNF-α stimulated cells was significantly enhanced in both cell lines by TNF-α treatment (Figure 3D). Given the direct involvement of IL-6 in PCa progression (25), we performed two functional assays to investigate the effect of TNF-α and IFN-γ on the ability of PCa cells to migrate and invade. In our experimental conditions, CA-HPV-10 showed very limited migratory capability, which was unaffected by either cytokine, whereas PC3 showed a constitutively high motility, which was significantly enhanced by TNF-α (12 h, *p* < 0.01; 24 h, *p* < 0.001) (Figures 3E,F). In addition, we observed that TNF-α strongly increased the invasiveness of PC3 by 53% (*p* = 0.03) and the invasiveness of CA-HPV-10 to a lesser extent by 32% (*p* = 0.04), when compared with IFN-γ-treated (PC3 24% and CA-HPV-10 8%) or -untreated cells (Figure 3G). Taken together these data indicate that inflammatory cytokines potentially present in PCa microenvironment are able to upregulate the expression of a restricted number of genes encoding factors of interest in PCa immunobiology.

### IFN-γ and TNF-α Activate JAK1/2 and p38MAPK Pathways in PCa, Respectively

**Figure 4:**
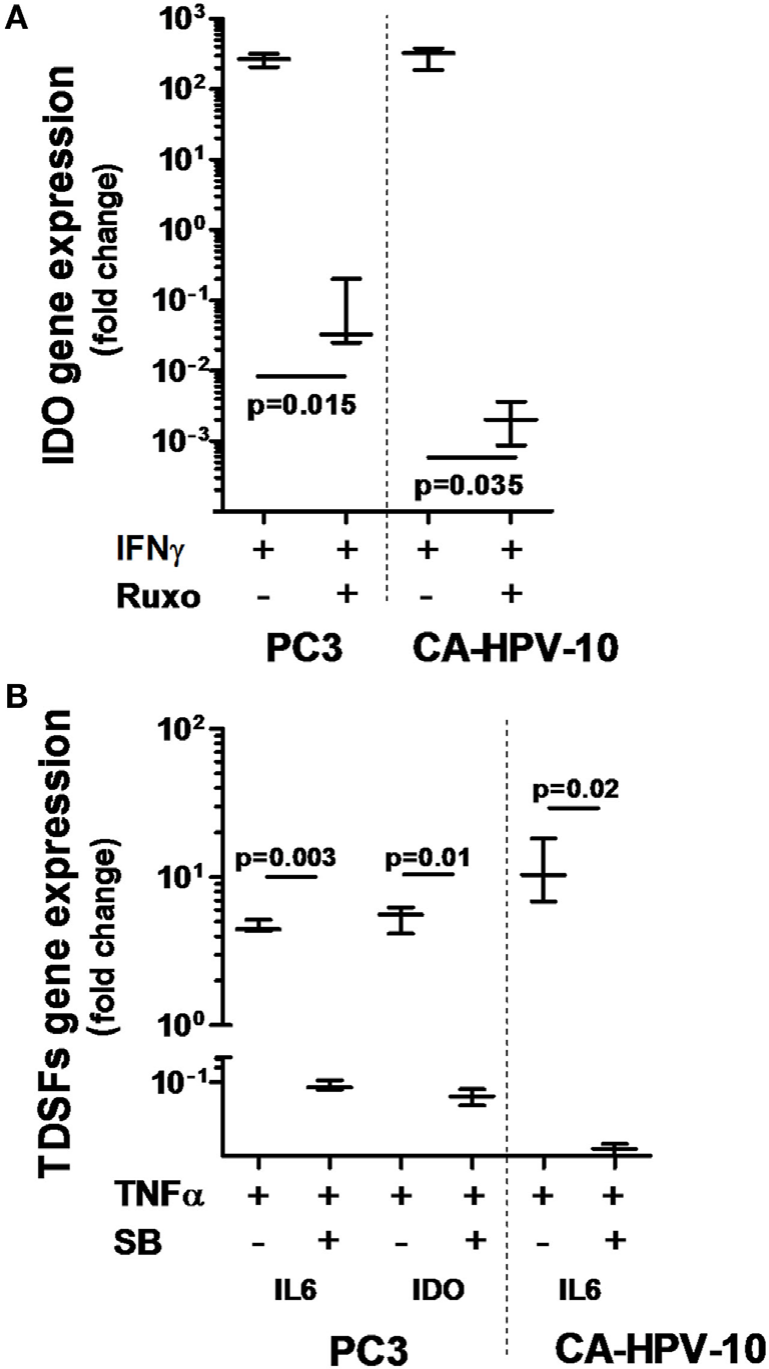
Interferon gamma receptor 1, 2 and tumor necrosis factor receptor 1 involvement in IFN-γ- and TNF-α-dependent indoleamine 2,3-dioxygenase (IDO) and interleukin (IL)-6 production. **(A)** IDO gene expression upon IFN-γ stimulation was significantly reduced by inhibition of the JAK-STAT signaling pathway with ruxolitinib in both cell lines tested (about 4-log less in PC3 and 5-log less in CA-HPV-10 cell lines). **(B)** The expression of IL-6 and IDO genes upon TNF-α stimulation was significantly reduced by inhibition of the p38MAPK kinase signaling pathway with SB202190 in both cell lines (Mann–Whitney *U* test). Experiments were conducted in triplicate and data are shown as box and whisker plot with SDs.

To confirm the ability of IFN-γ and TNF-α to stimulate PCa cells, we investigated the ability of well-characterized inhibitors of their signal transduction pathways to prevent their effects. Inhibition of the JAK1/2 pathway by ruxolitinib dramatically reduced IDO gene expression induced by IFN-γ in both PC3 (*p* = 0.02) and CA-HPV10 (*p* = 0.04) cells (Figure 4A). In contrast, NF-kB, p38MAPK, and PI3K inhibitors were ineffective (data not shown). Instead, inhibition of the p38MAPK pathway by SB202190 markedly reduced TNF-α-induced expression of IL-6 (*p* < 0.01), and IDO (*p* = 0.01) genes in PC3 cells and of IL-6 (*p* = 0.02) gene in CA-HPV10 cells (Figure 4B). However, inhibition of the NF-kB and PI3K pathways with PDTC and LY294992, respectively, did not significantly affect TNF-α stimulation in either cell lines (data not shown), suggesting a hierarchy of signal transduction checkpoints.

### Gene Expression Profiling in Clinical Specimens

“*In vitro*” data indicated that IFN-γ and TNF-α were able to enhance expression of IDO and IL-6 at the gene and protein level in PCa cells. Based on this background, we explored the expression of genes encoding these factors and their correlations in surgical BPH and PCa specimens.

**Figure 5:**
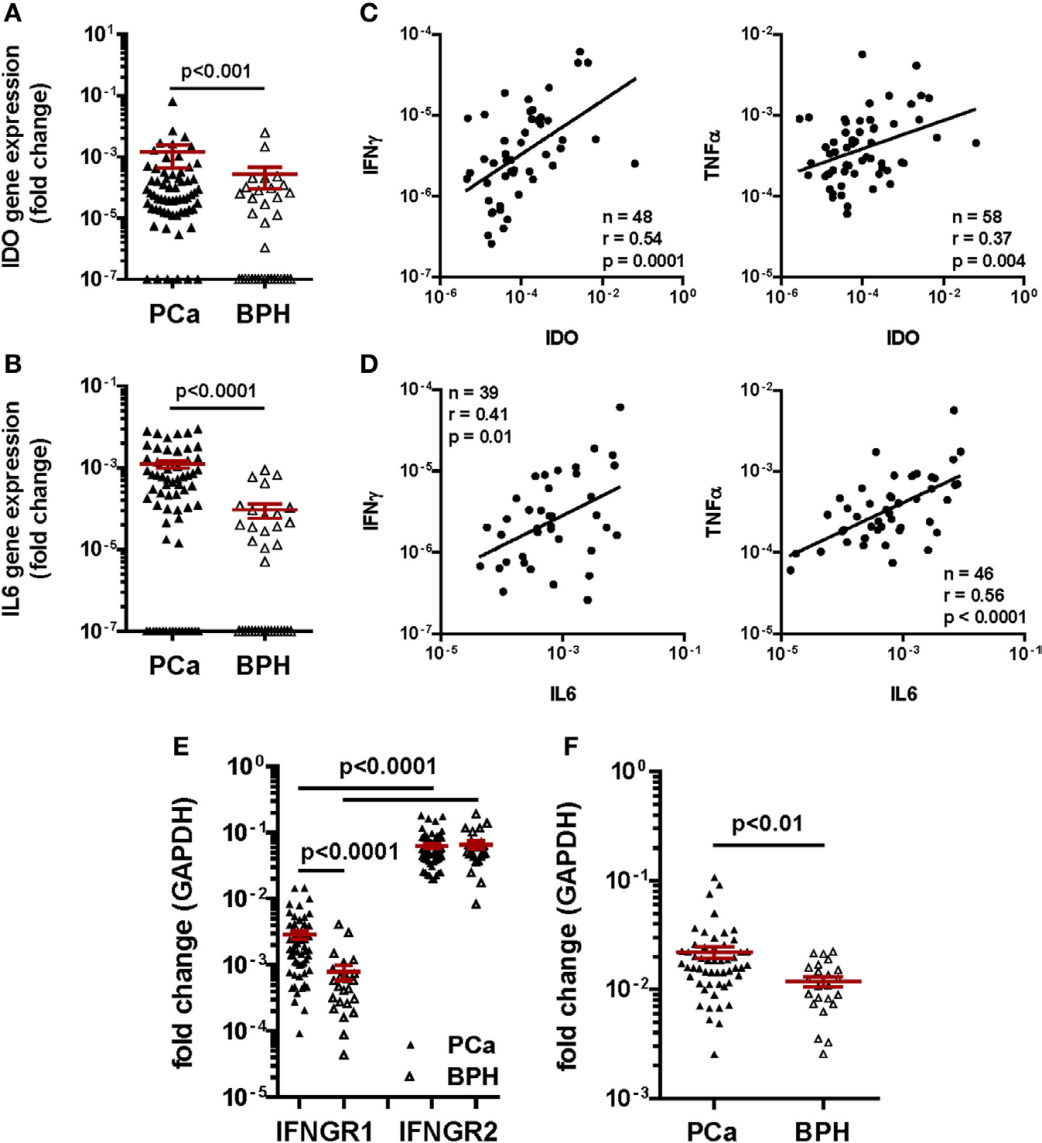
Gene expression profiling in clinical specimens. Gene expression of **(A)** Indoleamine 2,3-dioxygenase (IDO) and **(B)** interleukin (IL)-6 genes in prostate cancer (PCa) (black triangles) and benign prostatic hyperplasia (BPH) (open triangles). A higher expression of IDO and of both cytokine genes was detected in PCa than in BPH tissue specimens (Mann–Whitney *U* test; IDO *p* < 0.001, IL-6 *p* < 0.0001). The 2^−ΔΔ^*^Ct^* method was used to compute fold changes in gene expression for both cytokines, setting GAPDH gene expression equal to 1. Values ≤10^7^ were considered undetectable. **(C,D)** Fold changes in **(C)** IDO and **(D)** IL-6 gene expression in PCa tissue specimens were plotted against fold changes in IFN-γ (left panels) or TNF-α (right panels) gene expression in same specimens. A significant correlation was observed in all panels (Spearman rank correlation coefficient). **(E)** Gene expression of interferon gamma receptor 1, 2 (IFNGR1 and 2) in PCa (black triangles) and BPH (open triangles). A significantly higher expression of IFNGR2 than of IFNGR1 in both BPH and PCa tissue specimens was observed (Mann–Whitney *U* test, *p* < 0.0001). IFNGR1 gene was significantly more expressed in PCa than in BPH (Mann–Whitney *U* test, *p* < 0.0001). The 2^−ΔΔ^*^Ct^* method was used to compute fold changes in gene expression in both receptors, setting GAPDH gene expression equal to 1. Values ≤10^7^ were considered undetectable. **(F)** Gene expression of tumor necrosis factor receptor 1 in PCa (black triangles) and BPH (open triangles). A significantly higher expression of the receptor was observed in PCa than in BPH tissue specimens (Mann–Whitney *U* test, *p* < 0.01). The 2^−ΔΔ^*^Ct^* method was used to compute fold changes in gene expression in both receptors, setting GAPDH gene expression equal to 1. Values ≤10^7^ were considered undetectable.

Consistent with previous reports from our group (9), we observed that the frequency of detection of IDO (91% in PCa vs. 51% in BPH, *p* < 0.0001) and IL-6 (73% in PCa vs. 51% in BPH, *p* = 0.03) gene expression were significantly higher in PCa than in BPH tissues (Table 1). Similarly, their expression levels (IDO: *p* < 0.001 and IL-6: *p* < 0.0001) were significantly higher in PCa than in BPH tissues (Figures 5A,B).

Most interestingly, in PCa patients, IDO gene expression levels appeared to highly significantly correlate with those of IFN-γ (*n* = 48, *r* = 0.54; *p* < 0.001), and, to a lower extent, of TNF-α gene (*n* = 58, *r* = 0.37; *p* < 0.01) (Figure 5C). Intriguingly, but consistent with our “*in vitro*” data, levels of IL-6 gene expression correlated more significantly with those of TNF-α (*n* = 46, *r* = 0.56; *p* < 0.0001) than with those of IFN-γ (*n* = 39, *r* = 0.41; *p* < 0.01) gene expression (Figure 5D).

Prompted by these data, we also analyzed the expression of genes encoding IFN-γ and TNF-α receptors in clinical specimens. In our cohort of PCa patients, IFNGR2 gene expression was similar in PCa and BPH tissues. In contrast, expression of IFNGR1 gene, encoding the cytokine binding chain of the receptor, was significantly higher in PCa than in BPH tissues (*p* < 0.0001) (Figure 5E). Notably, TNFR1 gene also appeared to be expressed to significantly higher extents in PCa than in BPH specimens (*p* < 0.01) (Figure 5F).

### IDO^high^ Patients Are at Higher Risk of Developing BR in PCa

Our “*in vitro*” results indicated that IFN-γ and TNF-α were able to enhance the expression of IDO and IL-6 at gene and protein level in PCa cells. Furthermore, “*ex vivo*” data documented that both IDO and IL-6 genes are expressed to higher extents in PCa than in BPH, and that their expression is highly significantly correlated with that of IFN-γ and TNF-α genes. Taken together these results urged us to address the prognostic significance of IDO and IL-6 gene expression in 97 PCa, following RP.

**Figure 6:**
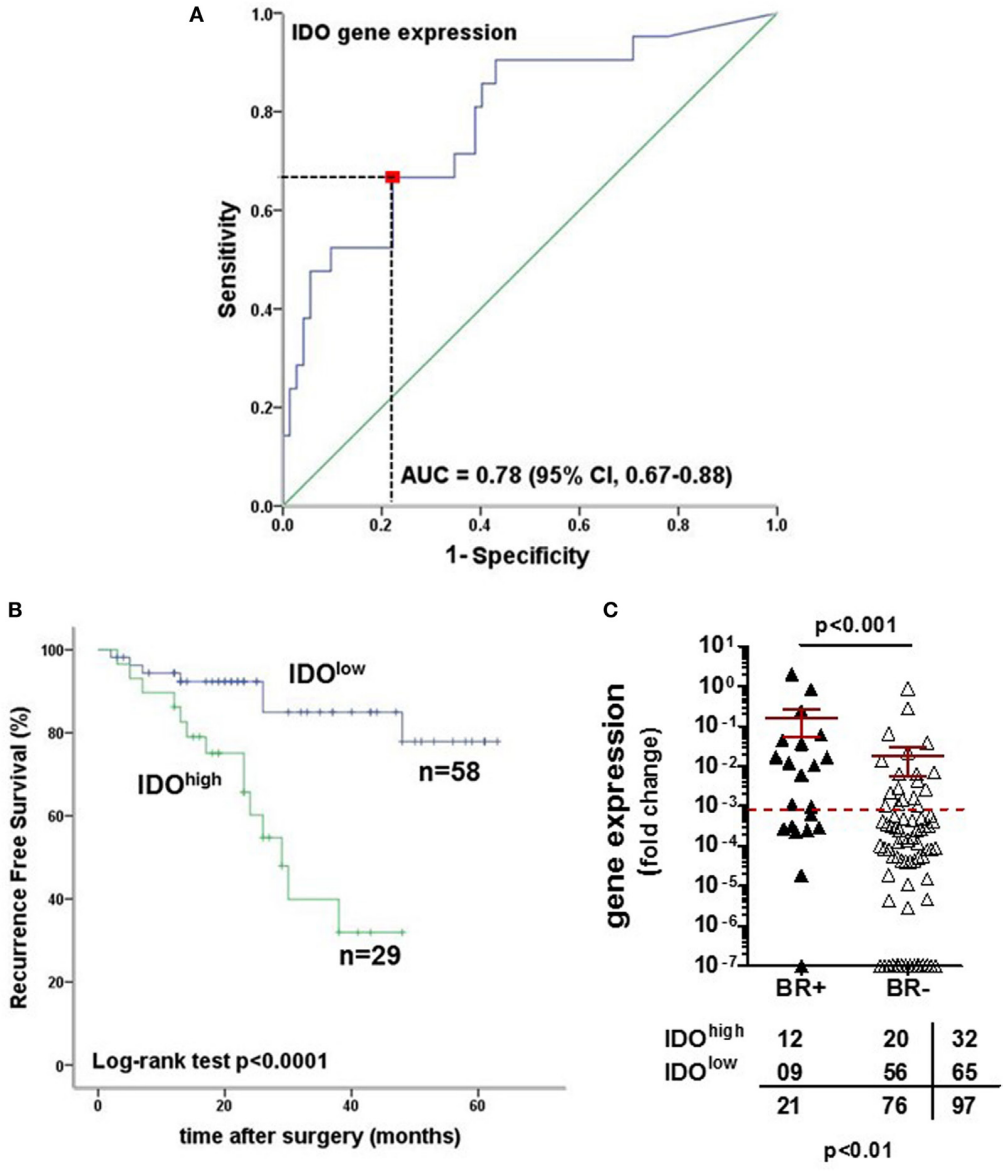
Indoleamine 2,3-dioxygenase (IDO) tumor-specific expression is a strong prognostic factor for biochemical recurrence (BR) in prostate cancer (PCa). **(A)** Receiver operating characteristic (ROC) curve analysis was used to evaluate the prognostic relevance of IDO gene expression. The area under the curve for recurrence-free survival (RFS) was 0.78 (95% CI, 0.67–0.88%) with an optimal cutoff level of 9.83 × 10^−4^ to dichotomize the patients into IDO^high^ and IDO^low^ groups (specificity 78%, sensitivity 67%). **(B)** Kaplan–Meier estimates of RFS stratified by the established cutoff for IDO gene expression in PCa tissue specimens (IDO 9.83 × 10^−4^). The survival analysis was based on time of BR (RFS with a median survival of 29 months; 95% CI, 22–36%; *p* < 0.0001, two-sided log rank test). **(C)** IDO gene expression in PCa tissue specimens was higher in BR-positive than in BR-negative patients (*p* < 0.001, Mann–Whitney *U* test), with a clear association between IDO gene levels and evidence of BR (prostate-specific antigen ≥ 0.1 ng/ml) (Pearson χ^2^ test).

We focused our analysis on the timing of so-called BR, as defined by PSA values ≥0.1 ng/ml. The prognostic relevance of the genes under investigation was evaluated using receiver operating characteristic (ROC) curve analysis. For IDO, the calculated area under the curve (AUC) for RFS was 0.78 (95% CI, 0.67–0.88%) (Figure 6A). ROC analysis for RFS defined an optimal cutoff level of 9.83 × 10^−4^ to dichotomize the patients into IDO^high^ and IDO^low^ groups (specificity 78%, sensitivity 67%). Estimated rate of 5-year RFS was 32% (95% CI, 26–38%, SE = 3.02) for IDO^high^ patients and 55% (95% CI, 49–60%; SE = 2.8; *p* < 0.001) for IDO^low^patients (Figure 6B). Overall, BR + patients showed a significantly higher expression of IDO gene than BR− patients (*p* < 0.001, Figure 6C). Cox regression analysis indicated that IDO^high^ PCa patients had a higher risk of BR than did IDO^low^ PCa patients (HR 3.1, *p* < 0.01, Table 3). In multivariate analysis, IDO^high^ PCa patients maintained a significant higher risk of BR than IDO^low^ PCa patients, irrespective of established predictors (Table 4).

**Table 3:** Univariate Cox regression analysis of the risk of biochemical recurrence associated with indoleamine 2,3-dioxygenase (IDO) and interleukin (IL)-6 gene expression.

Variable	Parameter	HR	95% CI	*p*-Value
IDO gene expression	IDO^high^ vs. IDO^low^	3.11	1.3–7.41	<0.01
IL-6 gene expression	IL-6^high^ vs. IL-6^low^	0.87	0.39–1.96	0.7

*HR, hazard ratio*.

*All p-values were two-sided*.

**Table 4:** Multivariate Cox regression analysis of the risk of biochemical recurrence (BR) associated with indoleamine 2,3-dioxygenase (IDO) gene expression level stratification adjusted for tumor stage (>3 vs ≥3) and Gleason score (≥6 vs ≥7).

Variable	Parameter	HR	95% CI	*p*-Value
IDO gene expression	IDO^high^ vs. IDO^low^	2.97	1.22–7.24	0.002
Gleason score	≥5–6 vs. ≤ 7–9	1.25	0.47–3.3	0.7
Tumor stage	pT ≥ 3 vs. pT < 3	1.51	0.41–5.61	0.5

*HR, hazard ratio*.

*All p-values were two-sided*.

For IL-6, 95% CI of AUC persistently included the 0.50 limit, consistent with a lack of prognostic potential. Indeed, neither a significant association between IL-6 gene expression levels and BR nor a significant impact on RFS were detectable (Figure S1 in Supplementary Material).

Contingency table analysis did not provide any evidence for an association between IDO gene expression in tumor specimens and three classical clinicopathological parameters, PSA, pT, and Gleason score, while IL-6 gene expression was associated with PSA (*p* = 0.02) and showed a trend toward association with Gleason score (*p* = 0.06) (Table 5).

**Table 5:** Distribution of clinical–pathological characteristics, in cancer sorted according to indoleamine 2,3-dioxygenase (IDO) or interleukin (IL)-6 gene expression levels.

Factors						
Characteristics	IDO^high^,*n* = 26	IDO^low^,*n* = 71	*p*-Value	IL-6^high^,*n* = 47	IL-6^low^,*n* = 50	*p*-Value^a^
**PSA at diagnosis**						
<10 ng/mL	16	51	1	36	31	0.02
>10 ng/mL	6	20		7	19	
Missing	0	4		4	0	
**Tumor stage**						
<pT3	5	16	1	13	7	0.2
>pT3	15	49		30	35	
Missing	3	9		4	8	
**Gleason score**						
≤7 (3 + 4)	9	25	0.6	21	13	0.06
≥7 (4 + 3)	13	50		26	37	
Missing	0	0		0	0	

*^a^Two-sided Fisher’s exact test*.

### IDO as Diagnostic and Prognostic Tool When Detected at Gene Level in Urine of Men at Risk of Developing PCa

**Figure 7:**
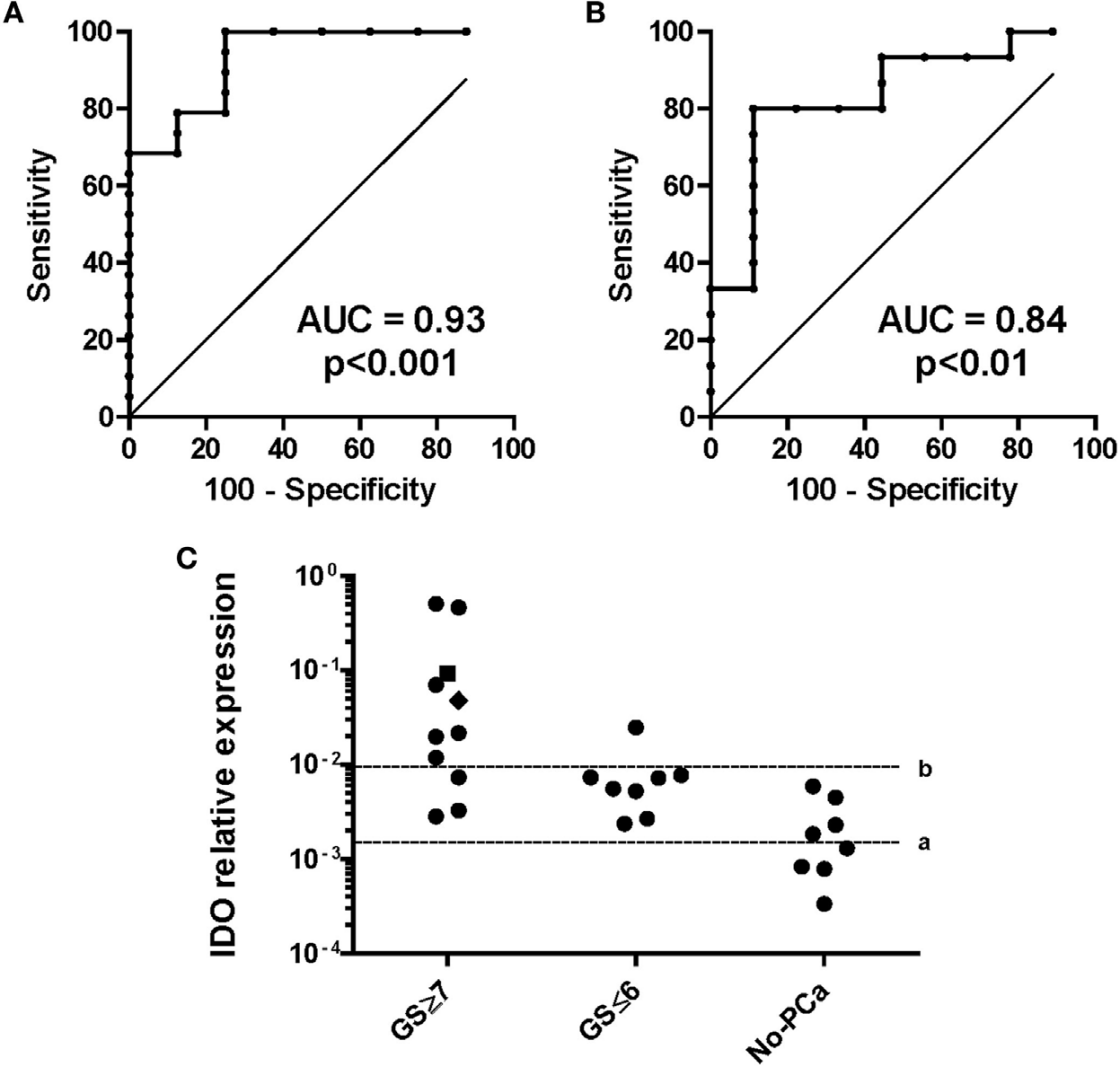
Indoleamine 2,3-dioxygenase (IDO) gene expression in urine sediments of individuals at risk of developing prostate cancer (PCa). IDO relative gene expression was analyzed in urine sediments from patients collected without previous manipulation of the prostate. **(A)** Definition of the thresholds for IDO mRNA for the probability of having PCa. The area under the curve (AUC) for the presence/absence of PCa was 0.93 (95% CI, 0.84–1.03%) with an optimal cutoff level of 0.0015 to dichotomize the patients into PCa positive and PCa negative (specificity 100%, sensitivity 75%). **(B)** Definition of the thresholds for IDO mRNA for the probability of having a clinically relevant (GS ≥ 7) than an indolent (GS ≤ 6) PCa. GS was evaluated from the biopsy or after prostatectomy, when available. The AUC for indolent/aggressive PCa was 0.84 (95% CI, 0.66–1%) with an optimal cutoff level of 0.0095 (specificity 89%, sensitivity 80%). **(C)** Cutoff a (≤0.0015), assignment to biopsy for the risk of having PCa (NPV = 100%, PPV = 82%). Cutoff b (≥0.0096), assignment to biopsy for the risk of having clinically relevant PCa (NPV 83%, PPV 88%). A value of 0.0479 (black square) represents the risk of having biochemical recurrence within 5 years from prostatectomy.

Capitalizing on data from surgical specimens, we hypothesized that IDO might represent a novel biomarker for PCa in urine. We studied 27 patients undergoing first time prostate biopsy for being at risk of PCa (based on positive digital rectal examination (DRE) and PSA serum level >4 ng/ml). Nineteen out of 27 prostate biopsies were positive for PCa (localized PC), while 4 were BPH and 4 were negative (normal tissue). Our data show that the quantification of IDO mRNA in urine of patients before undergoing biopsy for diagnostic procedures has a very promising ability to identify those harboring PCa. The AUC to dichotomize individuals based on the presence or absence of cancer defined a cutoff level of 0.0015 (Figure 7A). Therefore, with IDO ≤ 0.0015 gene expression levels, PCa risk is negligible (NPV 100%, PPV 82%). The AUC to dichotomize patients with indolent or aggressive PCa based on GS defined a cutoff level of 0.0096 (Figure 7B). It says that 87.5% of patients with indolent PCa (GS ≤ 6 and BR− and no treatment after biopsy), 100% of patients with no PCa, and 27% of patients with clinically relevant PCa (GS ≥ 7, BR−, treatment after biopsy) expressed IDO below 0.0096 (73% of patients with clinically relevant PCa above 0.0096; NPV 83%, PPV 88%) (Figure 7C). Overall, the IDO test might reduce the number of unnecessary biopsies by 14.8% with cutoff 0.0015 and 66.6% with cutoff 0.0096. Detection of IL-6 in urines of same individuals was neither effective in predicting patients bearing PCa nor in identifying, among diagnosed PCa, those at risk of developing an aggressive tumor (data not shown).

## Discussion

Pre-existing chronic inflammation has repeatedly been reported to play a key role in PCa outgrowth and progression (4). However, underlying molecular mechanisms are largely unclear. In this study, we have utilized clinical specimens and established cell lines to obtain innovative insights into the relationship occurring between expression of IFN-γ and TNF-α pro-inflammatory cytokines in PCa tissues, and tumor progression.

Our data indicate that expression of genes encoding IFN-γ and TNF-α is detectable in both BPH and PCa specimens. Intriguingly, the expression of both genes is significantly higher in BPH than in PCa tissues (34). However, expression of specific receptors appears to be higher in PCa than in BPH. Thus, these cytokines, produced by a variety of interstitial inflammatory cells, might be highly effective within malignant tissues. Remarkably, expression of IFN-γ and TNF-α genes is significantly correlated only in PCa, suggesting common induction mechanisms.

We used an *in vitro* model based on cell lines originating from localized, CA-HPV-10, and metastatic, PC3, disease to address direct effects of IFN-γ and TNF-α on PCa cells. Among a group of genes responsible for cell contact independent mechanism of tumor promotion and reportedly impacting on cancer progression (33), IDO and IL-6 genes were the only ones consistently overexpressed in PCa cells upon stimulation with IFN-γ or TNF-α. In particular, IFN-γ induced IDO gene upregulation in both cell lines under investigation, whereas TNF-α induced IL-6 gene overexpression in both cell lines and IDO gene overexpression in PC3 cells.

These findings, supported by ELISA and functional data have prompted us to investigate their clinical significance. *Ex vivo* results indicate that expression of IDO and IL-6 genes in PCa tissues is significantly correlated with IFN-γ and TNF-α gene expression. Most importantly, we observed that IDO, but not IL-6, gene expression predicts BR.

Taken together our data confirm and extend previous reports from our group (9). More interestingly, they unravel novel molecular mechanisms underlying the association between inflammation and PCa progression and suggest that cancer cell-specific molecular alterations (35) and concomitant inflammation (13), might independently contribute to give rise to highly aggressive PCa.

Indoleamine 2,3-dioxygenase is a tryptophan-depleting enzyme that has been thoroughly investigated by immunologists because of its involvement in the mechanisms of immune regulation (24). IDO blunts T lymphocyte expansion (36), thereby powerfully inhibiting adaptive immune responses. On the same line, more recently, IDO has been shown to induce resistance to treatment with therapeutic monoclonal antibodies targeting immunological checkpoints (37). These effects have been shown to be mediated, at least in part, by regulatory T cells-dependent recruitment and activation of myeloid-derived suppressor cells (38).

Most interestingly, in murine cancer models, high-IDO activity, resulting in accelerated tumor progression, is associated to immune responses and to high expression in the tumor microenvironment of pro-inflammatory cytokines, including IFN-γ and TNF-α (37). Notably, IFN-γ and TNF-α have previously been shown to powerfully synergize in the induction of IDO, although to different extents (39–42).

Exogenous administration of TNF-α also induced IL-6 release in both cell lines under investigation. IL-6 gene expression was also detected at significantly higher levels in PCa than in BPH specimens and was highly significantly correlated with TNF-α gene expression, but it did not impact on RFS. Indeed, IL-6 has been reported to contribute to the onset of castration-resistant PCa by activating alternative pathways involved in cancer survival and proliferation and by inducing androgen receptor overexpression in tumor cells (43). However, although IL-6 plasma levels have been suggested to predict severe prognosis (44–46), targeted treatments with therapeutic monoclonal antibodies have proved ineffective (47, 48).

Our results delineate a scenario where tumor cells may produce IDO, promoting the generation of an immune-suppressive microenvironment upon stimulation by pro-inflammatory cytokines, mainly IFN-γ. Therefore, while IDO would not “*per se*” induce PCa, it might favor its progression. Indeed, pro-inflammatory cytokines are also produced in conditions unrelated to neoplastic transformation. Interestingly, however, the involvement of IFN-γ in the development of prostate inflammation, such as prostatitis, is debated. Seminal plasma from patients with chronic prostatitis (CP)/chronic pelvic pain syndrome show significantly higher levels of TNF-α and IL-1β when compared with those from healthy subjects (49), while no involvement of IFN-γ has been reported (50, 51). Hence, the inflammatory status in CP appears to be mainly due to the involvement of TNF-α and the IL-1β/IL-6 axis (52). Our data suggest that TNF-α marginally contributes to modulate IDO gene expression patterns and enzymatic activity (39, 40).

Clarification of molecular mechanisms underlying inflammation-induced immune suppression might increase our ability to monitor the progression of PCa from a slow-growing, organ-confined tumor to a highly invasive, and castration-resistant malignancy. Our data from urine sediment of men undergoing first time fine needle biopsy for PCa diagnosis show that patients with elevated IDO gene expression levels are at high risk of harboring a clinically relevant form of PCa and have a higher probability of relapsing after prostatectomy. Despite the limited number of subjects screened, the strength of this pilot study is represented by its high sensitivity. None of the subjects found positive for the presence of PCa showed IDO gene expression level below the optimal cutoff level of 0.0015, as defined by ROC to dichotomize the patients into PCa positive and PCa negative (PPV 100%, no false negative). In addition, two patients negative for PCa with IDO gene expression levels in urine below the optimal cutoff of 0.0015 showed light inflammatory foci compatible with low-grade inflammation, as observed in biopsy material at histological level.

This novel screening approach based on the IDO mRNA levels in urine could offer innovative guiding tools for patient management thereby helping to avoid overtreatment. Furthermore, it would reduce the number of individuals undergoing prostate biopsy, as dictated by the low-sensitive PSA test. In addition, the test is quick, cost-effective, and it does not require DRE/prostate massage for reliable read-out in the urine sediment.

Limitations of our work should be acknowledged. In particular, our study largely relies on gene expression analysis and we could not address specific protein detection in clinical specimens. While gene expression does not always accurately reflect protein levels due to post-translational regulation, major obstacles for cytokine detection by immunohistochemistry are represented by the low sensitivity of available reagents and the frequently diffuse staining. In contrast, qRT-PCR is straightforward and allows detection of the expression of multiple cytokine genes from same specimen (53, 54). On a similar line, IDO protein detection is challenging since most specific commercial reagents are characterized by a relatively low sensitivity. Therefore, target protein is usually only observed in the presence of high levels of gene expression, as described in a previous report (9). However, our data indicate that tryptophan/kynurenine ratios are dramatically decreased in supernatants of cytokine-stimulated PCa cells, consistent with the production of functionally active enzyme (Figure 3C). Furthermore, most interestingly, recent studies, taking advantage of newly developed, non-commercially available reagents, clearly indicate that IDO protein expression is detectable in tumor cells in 20% of clinical PCa specimens (55).

On the other hand, while correlations between the expression of the genes under investigation and clinical course were revealed by our study, mechanistic aspects remain largely unclear and warrant further research taking advantage of updated experimental models and of the availability more effective reagents.

Analysis of larger cohorts of patients will also be needed to validate the clinical relevance of our data. Interestingly, the analysis of a larger cohort of about 500 samples, although provisional (TCGA; www.cbioportl.org), is in contrast with our finding concerning the association between high-IDO expression and survival of PCa. However, a critical requirement will be represented by a strict standardization of patient treatment and sample collection. Indeed, TCGA databases are generated upon evaluation of specimens from more heterogeneous cohorts of patients, undergoing a variety of different therapeutic procedures. Cases considered in our report were all from patients treated by the same group of urologists according to standardized procedures, including indications and timing for surgery and follow-up. Moreover, our gene expression data were analyzed by using specific ROC curves deriving from the combined analysis of qRT-PCR tests performed in a single lab and clinical results. In addition, TCGA data were obtained by RNA seq, a less sensitive technique, when compared with qRT-PCR.

A major problem in PCa treatment is represented by the individuation of patients requiring more aggressive therapeutic approaches, while avoiding overtreatment of the others. Therefore, the characterization of predictive PCa markers is of paramount clinical importance. Within this context, our results identify IDO as a predictor of poor prognosis in PCa, and the corresponding protein as a potential therapeutic target.

## Ethics Statement

This study was carried out in accordance with the recommendations of Ethical Committee of Basel-Stadt and Basel-Land (EKBB, Ref. Nr. EK: 176/07) and Ethic Committee of Zurich (ZH-NR: 2010-0081/0). The protocol was approved by the Ethical Committee of Basel-Stadt and Basel-Land and Ethic Committee of Zurich. All subjects gave written informed consent in accordance with the Declaration of Helsinki.

## Author Contributions

IB, GCS, and MP conceived the study. IB, CM, GM, AC, RB, GS, TSS, and GC performed the experiments. IB, CM, AC, GS, TSS, and GC analyzed the data. SW, TH, and MP performed sample collection. SW, TH, AB, and TS arranged clinical aspect. IB, CM, GCS, and MP wrote the paper. GCS and MP supervised the project.

## Conflict of Interest Statement

The authors declare that the research was conducted in the absence of any commercial or financial relationships that could be construed as a potential conflict of interest.

## Supplementary Material

Figure S1 Prognostic value of IL-6 gene expression in tumor samples. **(A)** Receiver operating characteristic (ROC) analysis to calculate the prognostic value of IL-6. The area under the curve (AUC) for recurrence-free survival (RFS) was 0.51 (95% CI, 0.37–0.65%) with no evidence for a prognostic potential. **(B)** Kaplan–Meier estimates of RFS stratified by an arbitrary cutoff (50th percentile) for IL-6 gene expression in PCa tissue specimens. The survival analysis was based on the time of BR. The estimated rate of 5-year RFS was 53% (95% CI, 48–58%; SE 2.7%) for IL-6^high^ patients and 50% (95% CI, 44–55%; SE 3.03%; *p* = 0.8, two-sided log rank test) for IL-6^low^ patients. **(C)** IL-6 gene expression in PCa tissue specimens was not significantly higher in BR-positive than in BR-negative patients (*p* = ns, Mann–Whitney *U* test). In addition, no association between IL-6 gene levels and evidence of BR (PSA ≥ 0.1 ng/ml) was documented (Pearson χ^2^ test).

## Abbreviations

PCa, prostate cancer; BPH, benign prostatic hyperplasia; TUR-P, transurethral resection of the prostate; CRPC, castration-resistant prostate cancer; BR, biochemical recurrence; PSA, prostate-specific antigen; TDSF, tumor-derived soluble factors; IDO, indoleamine 2, 3-dioxygenase; TNFR1, tumor necrosis factor receptor 1; IFNGR1, 2, interferon gamma receptor 1, 2; RFS, recurrence-free survival; AUC, area under the curve; ROC, receiver operating characteristic.
