# Supplementary figures and images for: Expression of Indoleamine 2,3-Dioxygenase Induced by IFN-γ and TNF-α as Potential Biomarker of Prostate Cancer Progression
Source: Front Immunol. 2018 May 29;9:1051. doi: 10.3389/fimmu.2018.01051 (PMC5986916; doi:10.3389/fimmu.2018.01051)

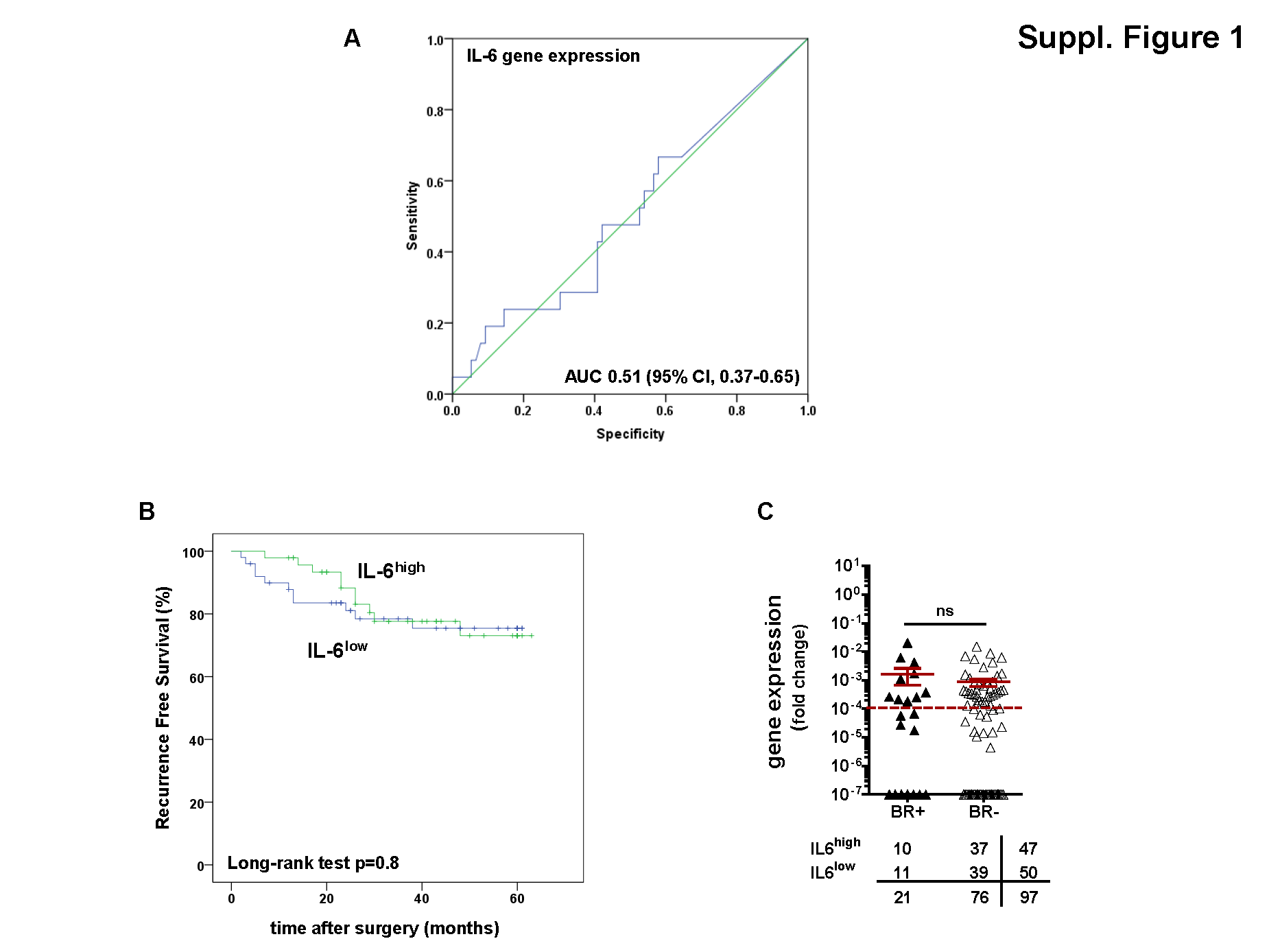

Supplement: Figure S1 — Prognostic value of IL-6 gene expression in tumor samples. (A) Receiver operating characteristic (ROC) analysis to calculate the prognostic value of IL-6. The area under the curve (AUC) for recurrence-free survival (RFS) was 0.51 (95% CI, 0.37–0.65%) with no evidence for a prognostic potential. (B) Kaplan–Meier estimates of RFS stratified by an arbitrary cutoff (50th percentile) for IL-6 gene expression in PCa tissue specimens. The survival analysis was based on the time of BR. The estimated rate of 5-year RFS was 53% (95% CI, 48–58%; SE 2.7%) for IL-6high patients and 50% (95% CI, 44–55%; SE 3.03%; p = 0.8, two-sided log rank test) for IL-6low patients. (C) IL-6 gene expression in PCa tissue specimens was not significantly higher in BR-positive than in BR-negative patients (p = ns, Mann–Whitney U test). In addition, no association between IL-6 gene levels and evidence of BR (PSA ≥ 0.1 ng/ml) was documented (Pearson χ2 test). [file image_1.tiff]
